# Supplementary material for: A Humanized Yeast Phenomic Model of Deoxycytidine Kinase to Predict Genetic Buffering of Nucleoside Analog Cytotoxicity
Source: Genes (Basel). 2019 Sep 30;10(10):770. doi: 10.3390/genes10100770 (PMC6826991; doi:10.3390/genes10100770)
Supplement: Supplementary file 1 [file genes-10-00770-s001.zip › Additional_Files/Additional_File7_GOTermHeatmaps.zip!/Additional_File7_GOTermHeatmaps/GDP-dissociation_inhibitor_activity.pdf]

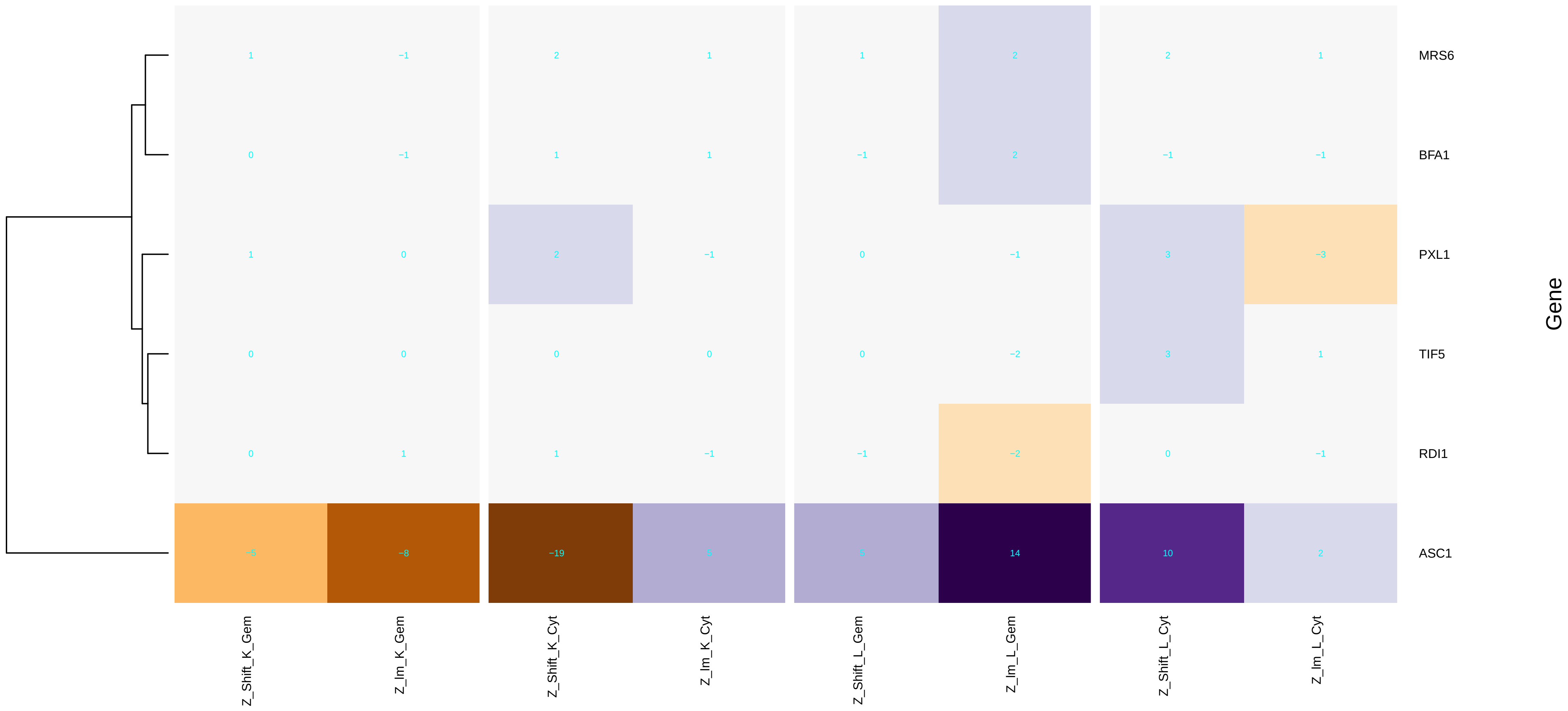

MRS6
1 −1 2 1 1 2 2 1
BFA1
0 −1 1 1 −1 2 −1 −1
PXL1
1 0 2 −1 0 −1 3 −3
TIF5
0 0 0 0 0 −2 3 1
RDI1
0 1 1 −1 −1 −2 0 −1
ASC1
−5 −8 −19 5 5 14 10 2
Z_Shift_K_Gem
Z_Im_K_Gem
Z_Shift_K_Cyt
Z_Im_K_Cyt
Z_Shift_L_Gem
Z_Im_L_Gem
Z_Shift_L_Cyt
Z_Im_L_Cyt
Gene
